# Microvascular invasion risk scores affect the estimation of early recurrence after resection in patients with hepatocellular carcinoma: a retrospective study

**DOI:** 10.1186/s12880-022-00940-0

**Published:** 2022-11-22

**Authors:** Sheng Wang, Weizhi Zheng, Zhencheng Zhang, Guo-hua Zhang, Dan-jiang Huang

**Affiliations:** 1 Department of Radiology, Taizhou First People’s Hospital, 218 Hengjie Rd., Dongcheng Street, Huangyan District, Taizhou City, 318020 Zhejiang Province China; 2 Department of Pathology, Taizhou First People’s Hospital, Taizhou City, 318020 Zhejiang Province China; 3 Department of Laboratory, Taizhou First People’s Hospital, Taizhou City, 318020 Zhejiang Province China

**Keywords:** Microvascular invasion, Hepatocellular carcinoma, Risk score, Recurrence

## Abstract

**Background:**

Microvascular invasion (MVI) is a histological factor that is closely related to the early recurrence of hepatocellular carcinoma (HCC) after resection. To investigate whether a noninvasive risk score system based on MVI status can be established to estimate early recurrence of HCC after resection.

**Methods:**

Between January 2018 to March 2021, a total of 108 patients with surgically treated single HCC was retrospectively included in our study. Fifty-one patients were pathologically confirmed with MVI and 57 patients were absent of MVI. Univariate and multivariate logistic regression analysis of preoperative laboratory and magnetic resonance imaging (MRI) features were used to screen noninvasive risk factors in association with MVI in HCC. Risk scores based on the odds ratio (OR) values of MVI-related risk factors were calculated to estimate the early recurrence after resection of HCC.

**Results:**

In multivariate logistic regression analysis, tumor size > 2 cm (*P* = 0.024, OR 3.05, 95% CI 1.19–11.13), Prothrombin induced by vitamin K absence-II > 32 mAU/ml (*P* = 0.001, OR 4.13, 95% CI 1.23–11.38), irregular tumor margin (*P* = 0.018, OR 3.10, 95% CI 1.16–8.31) and apparent diffusion coefficient value < 1007 × 10^− 3^mm^2^/s (*P* = 0.035, OR 2.27, 95% CI 1.14–7.71) were independent risk factors correlated to MVI in HCC. Risk scores of patients were calculated and were then categorized into high or low-risk levels. In multivariate cox survival analysis, only high-risk score of MVI was the independent risk factor of early recurrence (*P* = 0.009, OR 2.11, 95% CI 1.20–3.69), with a sensitivity and specificity of 0.52, 0.88, respectively.

**Conclusion:**

A risk score system based on MVI status can help stratify patients in high-risk of early recurrence after resection of HCC.

## Background

Hepatocellular carcinoma (HCC) is the most common liver cancer worldwide and the recurrence rate at 5 years after surgery for eligible patients with HCC is as high as 70% [1–3]. Early recurrence is currently considered as intrahepatic reoccurrence of primary HCC during the first two years after surgery and is associated with worse overall survival. The aggressiveness of several pathological factors of HCC, including low tumoral differentiation, microvascular invasion, is high-risk for early recurrence after resection [3–5].

Microvascular invasion (MVI) is one of the most important histological features that is closely related to postoperative early recurrence of HCC [6]. The treatments decision making may be changed for HCC patients with MVI [7, 8]. Though MVI can only be identified by histology, considerable efforts have been made to provide a noninvasive method to predict MVI status of HCC. Currently, α-fetoprotein (AFP) and Prothrombin induced by vitamin K absence-II (PIVKA-II) are important serum tumor markers in the detection of HCC [9–12]. The elevated serum level of PIVKA-II is associated with MVI and tumor recurrence [12]. The radiological methods including morphologic features and quantitative imaging parameters have been widely explored for predicting MVI status in HCC [13–18]. Some imaging features such as the tumor size, irregular shape, tumoral or peritumoral enhancement pattern, capsule appearance et al., are valuable characteristics of evaluating MVI. The further quantitative analyses of the radiomics or deep learning approaches are promising but technically complex in regarded as “black box” [19, 20]. Unfortunately, a reliable predictive model of MVI in consensus for estimating recurrence-free survival of patients after resection of HCC for an easier clinical use is still in lack.

Several previous studies have established a predictive model derived from MRI variables to directly estimate the risk of early recurrence after resection of HCC [21–24]. However, in these MR imaging-based models, the relationship between the risk factors and MVI was unclear, so these risk factors cannot replace the crucial role of pathologically determined MVI status for predicting early recurrence. Whether a predictive model of MVI can be used to predict postoperative early recurrence needs to be identified. We proposed that a simplified point scale can reflect the impact of each variable on MVI for patient risk stratification of early recurrence and thus benefit patients from therapeutic decision making.

In our study, we aimed to develop a noninvasive risk score system based on MVI to establish a predictive model for prognostic stratification of early recurrence in HCC patients after resection.

## Materials and methods

### Study population

**Fig. 1:**
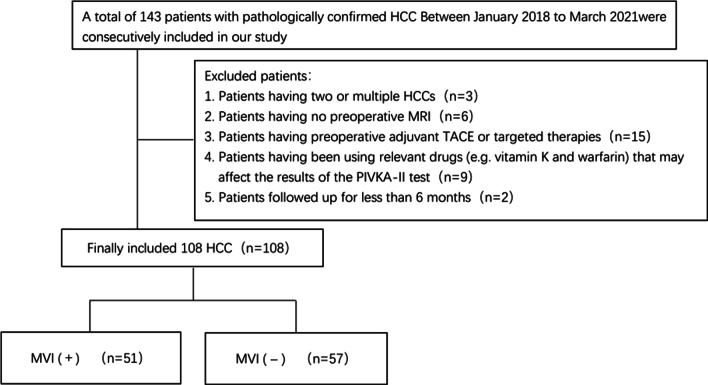
Flowchart of excluded patients in our study

This is a retrospective study with ethics committee approved by the local institutional review board (approval number 2020-KY002-01) and the requirement for written informed consent waived. Between January 2018 to March 2021, 143 patients in suspicious of having HCCs were consecutively included in our study, after reviewing the institutional radiological and histological database. Inclusion criteria: (1) pathological diagnosis of single HCC after surgery in confirmation with or without MVI; (2) having preoperative MRI and laboratory tests no more than 2 weeks before surgery; (3) receiving no preoperative adjuvant treatments. 35 patients were excluded for: (1) 3 patients having two or multiple HCCs; (2) 6 patients having no preoperative MRI; (3) 15 patients having preoperative adjuvant TACE or targeted therapies; (4) 9 patients having been using relevant drugs (e.g. vitamin K and warfarin) that may affect the results of the PIVKA-II test; (5) 2 patients followed up for less than 6 months (Fig. 1). Finally, a total of 108 patients with pathologically single HCC was included in our analysis, with 51 patients were presence of MVI and 57 were absence of MVI.

### Baseline characteristics and imaging variables

The baseline characteristics of patients including age, gender, etiology, alanine aminotransferase (ALT), aspartate aminotransferase (AST), serum alpha-fetoprotein (AFP), PIVKA-II level and maximum tumor diameter were recorded. The PIVKA-II level was measured on an instrument (ARCHITECT i 2000SR) by using the same batch of PIVKA-II test reagents manufactured by the company (Abbott, USA). A PIVKA-II level > 32 mAU/ml is considered positive.

First, two experienced radiologists in consensus evaluated the following MR imaging features: (1) the maximum tumor diameter, measured on the pre-contrast phase during dynamic contrast-enhanced MRI; (2) tumor margin, categorized as smooth and irregular tumor margin; (3) presence or absence of capsule enhancement; (4) apparent diffusion coefficient (ADC) values, measured three times at each section of the whole tumor, and the average value of the three times was calculated. Risk scores were simplified by rounding odds ratio (OR) values of risk factors of MVI to nearest half for establishing a predictive model of early recurrence. To test the reproducibility of the risk score system, the risk score of MVI for each HCC patient based on significant imaging features was assessed by a third radiologist independently for evaluating the inter-observer agreement.

### MRI protocol

All MRI examinations were performed using a German Siemens 1.5 T magnetic resonance scanner with a body phased array coil. The standard imaging protocol included transverse T1-weighted fat suppression imaging (repetition time (TR):132.00ms, echo time (TE):5.09ms, bandwidth (BW):179.00, flip angle (FA):60.00°, slice thickness: 6.00 mm, layer spacing: 1.80 mm), T2-weighted cross-sectional fat suppression imaging (TR:5500.18ms, TE:101.00ms, BW: 260.00, FA:140.00°, slice thickness:6.00 mm, layer spacing:1.80 mm), diffusion-weighted imaging (b = 800s/mm^2^) and apparent diffusion coefficient (ADC, TR:5574.23ms, TE:72.00ms, BW:1736.00, FA: 90.00, slice thickness:6.00 mm, layer spacing:1.80 mm). The contrast agent gadopentetate was injected intravenously at a rate of 2 ml/s at 0.2 mmol/kg and flushed with saline. Scans of arterial, portal and delayed phases were taken at 22s, 60 and 180 s post-injection, respectively.

### Pathological diagnosis of MVI

The “7-point” baseline sampling method was used, with 1:1 sampling of tumor and peritumoral liver tissue at 12, 3, 6 and 9 points of the tumor [7]. The pathological diagnosis report included description of the gross specimen, immunohistochemical staining, and MVI status. MVI was diagnosed as presence of nesting clusters of tumor cells in the portal vein branches, hepatic vein or endothelium-lined vasculature under the microscope.

### Follow-up

For HCC patients after resection, ultrasound and serum AFP test were performed every 3 months during the first 2 years and every 6 months thereafter during follow-up. All patients were follow-up for at least 6 months until the end of our study. Dynamic contrast-enhanced CT/MRI or ^18^ F-fluorodeoxyglucose positron emission tomography (PET-CT) would be performed for further evaluation if patients have suspicious recurrence detected on US or with elevated AFP during follow-up. The intrahepatic recurrence was identified by either pathological findings or typical imaging features of HCC [25, 26]. Early recurrence was defined as intrahepatic recurrence during the first 2 years after resection of HCC [3].

### Statistical analysis

Student’s t-test was used for comparison between normally distributed variables that expressed as mean ± standard deviation. Mann–Whitney U test were used for comparison between non-normally distributed variables that expressed as median with range in parentheses. Chi-square test was used for comparison between categorical groups. Univariate and multivariate logistic regression was applied to screen the independent risk factors of MVI and early recurrence. OR with 95% confidence interval (95% CI) were calculated. Inter-correlation coefficient (ICC) of risk scores was calculated between two observers. Risk scores with ICC value greater than 0.75 were considered of good reproducibility [27]. The receiver operating characteristic (ROC) curve of ADC value with area under ROC curve (AUC) and the cutoff value calculated. The sensitivity, specificity of risk score levels were calculated. P value < 0.05 was considered a statistically significant difference. SPSS software (version 21.0; SPSS, Chicago, Ill) was used for data analysis.

## Results

### Patient characteristics

A total of 108 HCC patients (94 males and 14 females) were included in our study. Among the 108 patients, 51 patients were presence of MVI and 58 patients were absence of MVI, with an average age of 61 years. The mean follow-up time of all 108 HCC patients was 16.9 ± 9.1 months (range: 6–36 months). During follow-up, 46 (42.6%) patients had early recurrence of HCC after resection, with an average time to early recurrence of 13.3 ± 6.5 months.

.

### Risk factors of MVI

The demographic, laboratory and imaging characteristics were demonstrated in Table 1. The results showed that tumor diameter, serum PIVKA-II level, tumor shape and ADC values were significantly different between MVI positive and negative groups. The AUC of ADC value for predicting MVI in HCC is 0.849 (0.776–0.922) with a cut-off value of 1007 × 10^− 3^mm^2^/s. In univariate analysis, the risk factor of tumor size > 2 cm, PIVKA-II > 32 mAU/ml, irregular shape, ADC < 1007 × 10^− 3^mm^2^ and capsule enhancement were associated with MVI in HCC patients (all *P* < 0.05). In multivariate analysis, tumor size > 2 cm (*P* = 0.024, OR 3.05, 95% CI 1.19–11.13), PIVKA-II > 32 mAU/ml (*P* = 0.001, OR 4.13, 95% CI 0.23–11.38), irregular shape (*P* = 0.018, OR 3.10, 95% CI 1.16–8.31) and ADC < 1007 × 10^− 3 ^mm^2^ (*P* = 0.035, OR 2.27, 95% CI 1.14–7.71) were independent risk factors of MVI (Table 2). Table 3 demonstrated that the ICC of risk scores from significant imaging features in multivariate analysis was 0.881 (95% CI 0.830–0.917).

**Table 1:** Comparisons of baseline variables between MVI-positive and MVI-negative groups

Variable	MVI (+)	MVI (−)	*P* value
Age (years)	61 ± 11	61 ± 9	0.969
Sex			0.355
Male	46	48	
Female	5	9	
Etiology			0.867 **
Hepatitis B virus	47	53	
Hepatitis C virus	2	3	
None or other	2	1	
Aspartate aminotransferase (IU/L)	40.5 ± 30.7	33.5 ± 33.2	0.259
Alanine aminotransferase (IU/L)	39.7 ± 35.0	32.4 ± 38.9	0.310
Histological grade			0.642
Low	26	31	
High	21	30	
Tumor diameter (cm)	46.9 ± 28.6	23.3 ± 10.5	< 0.001
> 2 cm	34	10	< 0.001
≤ 2 cm	17	47	
Alpha-fetoprotein (ng/ml)	742.4 (1.5-27738.3)	172.7 (1.2-3634.5)	0.093*
≥ 20 ng/ml	22	20	0.433
< 20 ng/ml	29	37	
PIVKA-II (mAU/ml)	3526.4 (16.7-30000)	412.8 (12.1-14817)	< 0.001*
> 32 mAU/ml	40	18	< 0.001
≤ 32 mAU/ml	11	39	
Tumor shape			0.001
Irregular	29	15	
Smooth	22	42	
ADC (×10^− 3^ mm^2^/sec)	930.7 ± 102.3	1059.5 ± 127.4	< 0.001
Capsule enhancement			0.103
Presence	33	28	
Absence	18	29	

*P value were calculated by using Mann-Whitney U test

**Data were compared using the Fisher’s exact test

PIVKA-II: prothrombin induced by vitamin-K-absence-II; ADC: apparent diffusion coefficient

**Table 2:** Univariate and Multivariate analysis of risk scores for MVI in HCC.

	Univariate		Multivariate		
	OR (95% CI)	*P*	OR (95%CI)	*P*	Risk score
Age	0.98 (0.95–1.02)	0.337			
Sex	0.13 (0.02–1.15)	0.067			
Hepatitis B virus	1.02 (0.97–1.04)	0.967			
Size > 2 cm	9.40 (3.83–23.05)	< 0.001	3.05 (1.19–11.13)	0.024	3
Alpha-fetoprotein ≥ 20 ng/ml	1.40 (0.65–3.05)	0.392			
PIVKA-II >32 mAU/ml	7.88 (3.30-18.81)	< 0.001	4.13 (1.23–11.38)	0.001	4
Irregular Shape	3.69 (1.64–8.29)	0.002	3.10 (1.16–8.31)	0.018	3
ADC < 1007 × 10^− 3^mm^2^/s	3.97 (1.78–8.85)	0.001	2.27 (1.14–7.71)	0.035	2
Capsule enhancement	0.51 (0.36–0.93)	0.034	1.90 (0.88–4.12)	0.105	
Alanine aminotransferase	1.01 (0.94–1.02)	0.324			
Aspartate aminotransferase	1.01 (0.99–1.08)	0.279			

PIVKA-II: prothrombin induced by vitamin-K-absence-II; ADC: apparent diffusion coefficient; OR: odds ratio; 95% CI: 95% confidence interval

**Table 3:** Reproducibility in the evaluation of risk scores between two observers

	Observer 1	Observer 2	ICC (95%CI)
Number of patients			
Size > 2 cm	44	51	
PIVKA-II >32 mAU/ml	58	58	
Irregular Shape	44	47	
ADC < 1007 × 10^− 3^mm^2^/s	51	56	
High-risk of MVI (8–12 point)	38	40	
Low-risk of MVI (0–7 point)	70	68	
Risk score	5.5 ± 4.1	5.9 ± 3.9	0.881 (0.830–0.917)

ICC: Inter-correlation coefficient

### Prognostic stratification based on risk score

**Fig. 2:**
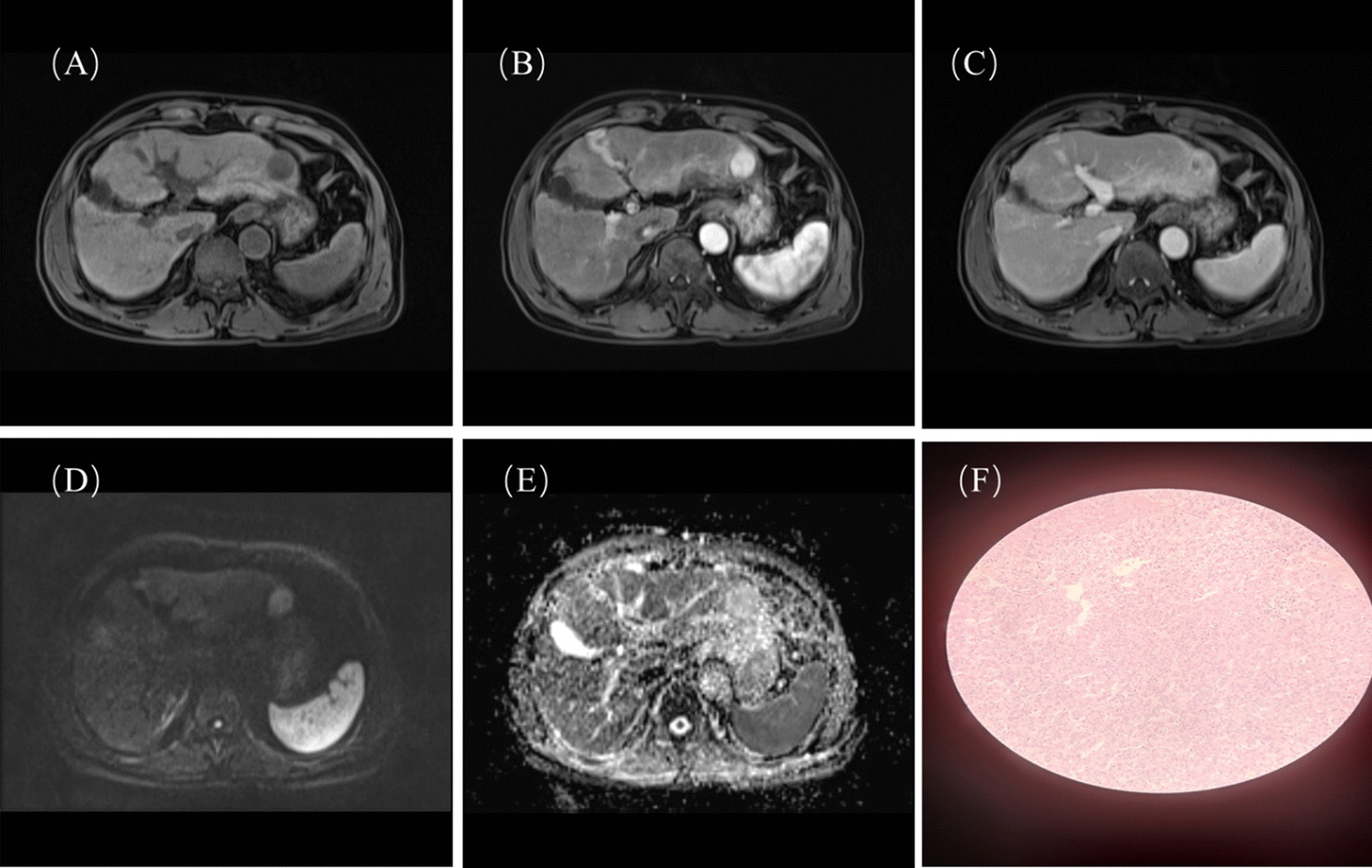
A 72-year-old patient with the HCC in the left lobe of liver were histologically confirmed of MVI negative status (risk score of 3 points) and did not have early recurrence during postoperative follow-up. **A** Pre-contrast phase; **B** Arterial phase; **C** Venous phase; **D** high b value map of DWI; **E** ADC map; **F** no MVI appearance under the microscope

**Fig. 3:**
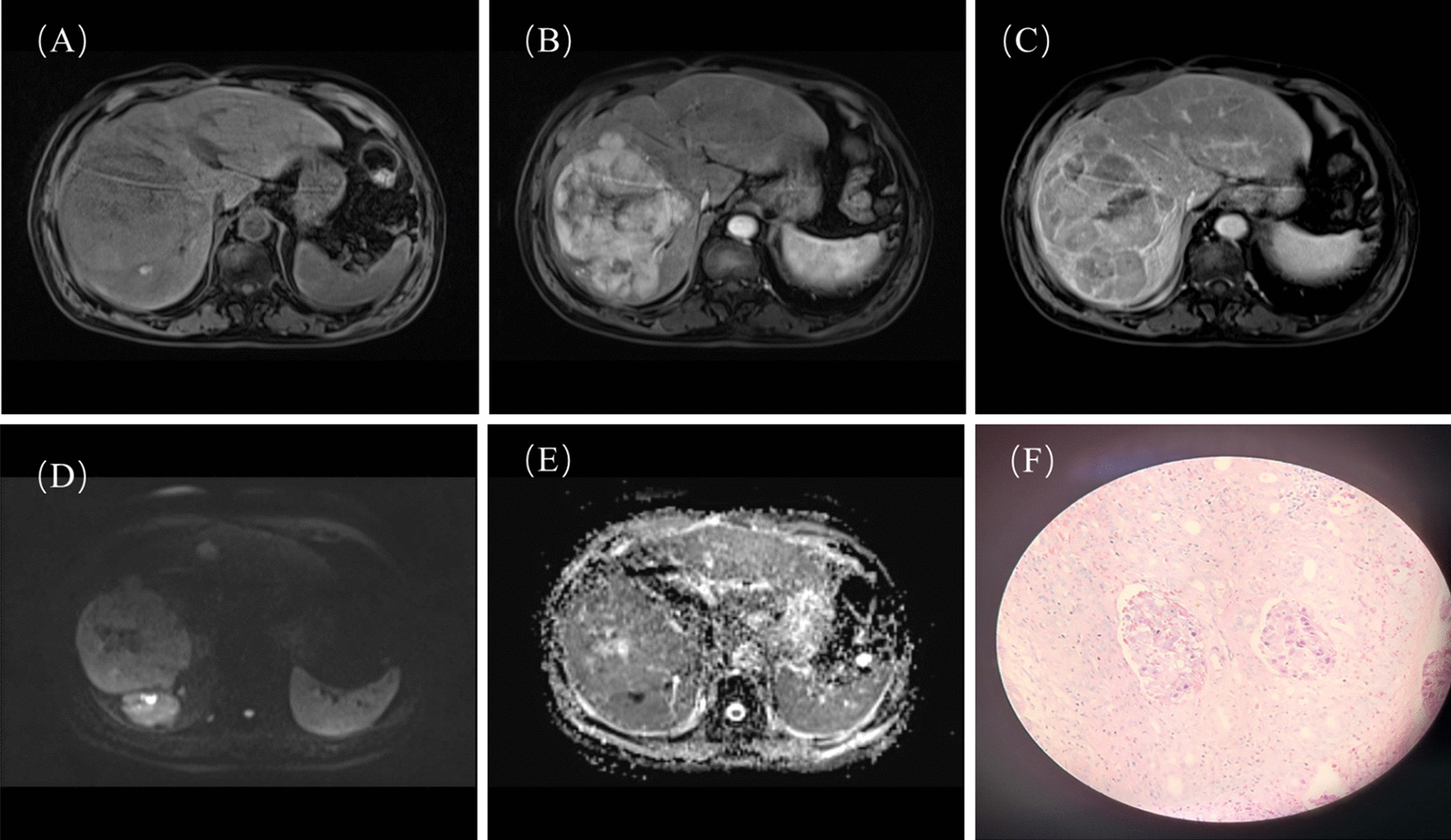
A 56-year-old patient with the HCC in the right lobe of liver were histologically confirmed of presence of MVI (risk score of 12 points) and had early recurrence in 8 months after resection. **A** Pre-contrast phase; **B** Arterial phase; **C** Venous phase; **D** high b value map of DWI; (E) ADC map; **F** presence of MVI under the microscope

**Fig. 4:**
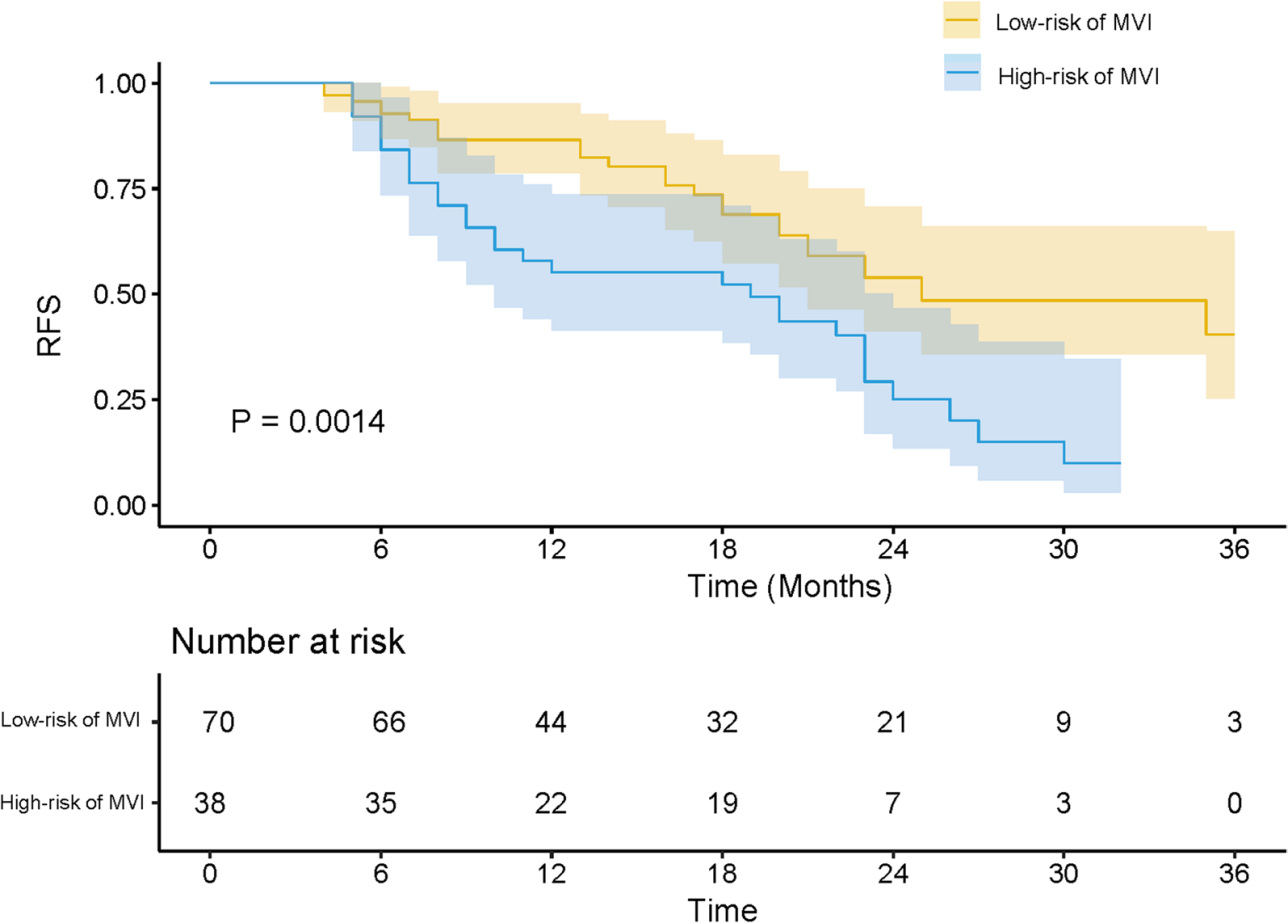
Recurrence-free survival curves (RFS) scaled by risk levels of MVI in the HCC patients

The risk score was weighted based on the OR values of MVI in multivariate analysis. The risk score of tumor size > 2 cm, PIVKA-II > 32 mAU/ml, irregular tumor margin and ADC < 1007 × 10^− 3^mm^2^/s was 3, 4, 3, 2 points, respectively. The risk factors of tumor size > 2 cm, PIVKA-II > 32.01 mAU/ml, ADC < 1007 × 10^− 3^mm^2^/s and high histological grade were significantly different between patients with or without early recurrence while irregular tumor shape showed no statistical significance (Table 4). The risk score ranged from 0 to 12 points. Patients were then categorized into low-risk (risk score: 0–7 points) and high-risk (risk score: 8–12 points) levels. The high or low-risk score level can significantly stratified prognostic difference of early recurrence (*P* < 0.001), with a relative low sensitivity of 0.52 but high specificity of 0.88 for estimating early recurrence. Among 108 patients of HCC, 70 patients had low-risk scores of MVI (Fig. 2) and 26 of them (26/70, 37.1%) had early recurrence; 38 patients had high-risk scores of MVI and 29 of them (29/38, 76.4%) had early recurrence (Fig. 3). In univariate cox survival analysis, tumor size > 2 cm, PIVKA-II>32 mAU/ml, ADC < 1007 × 10^− 3^mm^2^, high histological grade and high-risk score of MVI were statistically significant risk factors of early recurrence. In multivariate cox survival analysis, only high-risk score of MVI was independent risk factor of early recurrence (*P* = 0.009, OR 2.11, 95% CI 1.20–3.69) (Table 5). The Kaplan-Meier curves in Fig. 4 showed that the RFS in high-risk group of MVI was worse than low-risk group of MVI (*P* = 0.0014).

**Table 4:** Comparisons of MVI-related risk factors between presence and absence of early recurrence of HCC after resection

Risk factors	No. of patients	Early recurrence	No early recurrence	*P*
Tumor size > 2 cm	44	25	19	0.048
PIVKA-II >32.01 mAU/ml	58	33	25	0.010
Irregular Shape	44	22	22	0.439
ADC < 1007 × 10^− 3^mm^2^/s	51	31	20	0.002
High histological grade	47	31	16	0.006
Risk level of MVI*				< 0.001
High-risk (8–12 point)	38	29	9	
Low-risk (0–7 point)	70	26	44	

PIVKA-II: prothrombin induced by vitamin-K-absence-II;

ADC: apparent diffusion coefficient

**Table 5:** Cox survival analysis of MVI-related risk factors and scores for early recurrence of HCC after Resection

	Univariate		Multivariate	
	HR (95% CI)	*P*	HR (95%CI)	*P*
Size > 2 cm	1.74 (1.02–2.96)	0.041	1.30 (0.56–3.05)	0.539
PIVKA-II >32.01 mAU/ml	1.93 (1.08–3.46)	0.027	1.39 (0.62–3.12)	0.427
Irregular Shape	1.40 (0.81–2.41)	0.224		
ADC < 1007 × 10^− 3^mm^2^/s	1.95 (1.13–3.36)	0.016	1.71 (0.93–3.14)	0.082
High histological grade	1.82 (1.06–3.11)	0.028	1.34 (0.50–3.56)	0.504
High-risk score of MVI	2.30 (1.35–3.94)	0.002	2.11 (1.20–3.69)	0.009

PIVKA-II: prothrombin induced by vitamin-K-absence-II; ADC: apparent diffusion coefficient; HR: Hazard ratio

## Discussion

Our study developed a simple risk score based on MVI status by evaluation of preoperative radiological and laboratory characteristics. A high-risk score of MVI can provide a reliable prognostic stratification in early recurrence of HCC after resection.

In recent years, many scholars have paid attention to the noninvasive evaluation of MVI in HCC, because MVI is one of the most important pathological factors in association with aggressiveness of tumor and postoperative early recurrence.

Our study found that PIVKA-II-positive HCCs were more likely to have MVI than PIVKA-II-negative HCCs. PIVKA-II promotes proliferation of HCC cells and induces angiogenesis in surrounding liver tissue, thus promote vascular invasion of HCC [28, 29]. Previously, Yu et al. [15] found that the sensitivity of PIVKA-II in diagnosing HCC was higher than that of AFP, especially in early-stage HCC. The elevated level of PIVKA-II tests was associated with MVI in HCC [9, 12], which is in consistent with our findings. In our multivariate analysis, the PIVKA-II showed the highest OR value among the risk factors, indicating that PIVKA-II was the most important risk factor related to MVI in HCC.

Our risk score system contained some important imaging features in predicting MVI status of HCC. Consistent with previous reports, the larger tumor size and irregular tumor margin were proposed as significant risk factors that related to MVI in HCC. As the tumor size increases during progresses, the risk of MVI and intrahepatic metastasis increases. Pawlik et al. [16] investigated the relationship between the tumor size in 1073 HCC patients and MVI, and found that single HCC larger than 5 cm had significantly increased incidences of MVI. The tumor margin evaluated on MRI reflects the pathological characteristics of gross appearance. The gross types of “nodular with extranodular growth” and “multinodular confluent type” showing irregular tumor margin on MRI may have a higher incidence of MVI in HCC [30, 31]. The quantitative parameter of ADC value was included in our risk score because ADC reflects the extent of diffusion hindrance and mobility of water molecules and indicates tumor cellularity and microenvironment [32]. Previously, Suh et al. [14] found that an ADC value ≤ 1.11 × 10^− 3^mm^2^/s was an independent risk factor for predicting MVI with a high sensitivity and specificity of 93.5% and 72.2%, respectively. Xu et al. [15] reported that an ADC value ≤ 1.227 × 10^− 3^mm^2^/s (b = 500s/ mm^2^) was an independent risk factor for predicting MVI in HCC small than 2 cm, with a sensitivity and specificity of 66.7% and 78.6%, respectively. In our risk score, ADC showed the lowest OR values among the risk factors, the reason may be that ADC values vary widely during measurement and may not be adapted in generalization [33].

Many previous predictive models of MVI may have limited clinical utility because most models are not applicable to the prognostic stratification of early recurrence. Also, several predictive models of early recurrence have been proposed but the relationship between predictive factors of early recurrence and MVI may be lack of interpretation. Our proposed risk score based on MVI status is simple and easily to be evaluated, which may help clinicians to estimate the prognostic stratification of early recurrence after resection with a pathology-based explanation. Patients eligible for surgery with a high-risk score of MVI may have a higher rate of early recurrence so that the adjuvant therapy may be needed to be considered.

Our study has limitations. First, due to the small sample size in this retrospective study, selective bias cannot be avoided. Only solitary HCC was enrolled in our study. Second, some other risk factors of MVI were previously reported but were not included in our analysis. In the limited sample size, we selected some commonly used features that can be easily evaluated to facilitate a clinical use. Third, the risk score was established based on MVI status to provide a prognostic stratification of HCC patients. Whether there was potential intrahepatic metastasis or biliary infiltration indicating microinvasion in association with early recurrence was unclear and intraoperative ultrasound would be suggested for further evaluation [34]. Finally, our risk score is preliminary and needs further external validation with use of prospective cohort.

In conclusion, we developed a risk score system based on MVI status of HCC by integrating the noninvasive radiological and laboratory features to provide a stratification of patients in high-risk of early recurrence after resection, which is preliminary and needs further validation.

## Data Availability

The datasets generated during and analyzed during the current study are not publicly available due to protection of study participant privacy but are available from the corresponding author on reasonable request.

## Abbreviations

MVI: Microvascular invasion
HCC: Hepatocellular carcinoma
AFP: Alpha-fetoprotein
PIVKA-II: Prothrombin induced by vitamin K absence-II
ALT: Alanine aminotransferase
AST: Aspartate aminotransferase
OR: Odds ratio
PPV: Positive predictive value
NPV: Negative predictive value
ADC: Apparent diffusion coefficient
BW: Bandwidth
FA: Flip angle
